# Understood Consent Versus Informed Consent: A New Paradigm for Obtaining Consent for Pediatric Research Studies

**DOI:** 10.3389/fped.2013.00038

**Published:** 2013-11-21

**Authors:** Alan F. Isles

**Affiliations:** ^1^Royal Children’s Hospital, Brisbane, QLD, Australia; ^2^University of Queensland, Brisbane, QLD, Australia; ^3^Queensland Children’s Medical Research Institute, Brisbane, QLD, Australia

**Keywords:** informed consent, understood consent, readability, conflicts of interest, developing countries

## Abstract

All too often the informed consent process is viewed by members of research teams as a challenge of getting a parent or young person’s signature on a form. Informed consent is, however, much more than a signed form. Rather, it is a process, often iterative, in which the parent or young person is given sufficient information about a study in order that they can make a truly informed decision about participation. Substantial effort is required in producing appropriately formatted and readable documents using plain language at about Grade 6 or 12-year old reading level. Achieving truly understood consent involves the researcher spending significant one-on-one time with the parent or young person explaining in simple language what is proposed and then using so-called repeat-back techniques to test the understanding of the participants. This is critically important if the research involves randomization to different treatments or use of a placebo arm and, in particular if the research involves more than minimal risk.

Obtaining consent from a parent for their child to participate in a research study is often reduced to a process whereby the parent, or young person, is given an explanation of the study and an Information Package and the researcher or, more often, a member of the research team then collects a signature for participation on a consent form. While this description may sound somewhat extreme, it is reflective of how consent is frequently collected. I argue that consent, informed consent and understood consent have quite different meanings. The ideal goal of researchers recruiting subjects for a research study should be *understood consent*.

Prospective research participants may only understand 30–80% of the information in standard consent processes (1). As the goal of understood consent is currently rarely achieved, it is necessary to examine some of the contributing causes for this failure.

A major factor is that the focus of the consent process has changed from serving the needs of the patient to satisfying the medico-legal needs of institutions. Both institutions and researchers need to comply with regulatory requirements which now mandate the information that must be provided to a research subject. A consequence of this change in focus has been that Information/Consent packages for research studies have grown in both length and complexity. This involves a significant trade-off as it is well documented that as the length and complexity of an Information/Consent Package increases, the comprehension of the potential participant decreases (2). It can also take research staff significant time to explain these lengthy documents and, if there are time constraints, this can lead inadequate explanation and searching for understanding versus simply providing the information.

The readability of the Information Package is also critical. From the participant’s perspective, such documents need to be in plain language and it is recommended that they be written at a Grade 6 or 12-year old reading level (3–5). The regulatory and medico-legal needs of institutions typically win out over providing simple, plain language information that is likely to best meet the needs of the parent or participant.

## Pre-Conditions for Research on Children

There are two fundamental pre-conditions that must be satisfied for all research on children. Firstly, the research must be both scientifically and ethically sound. Secondly, a clinical research study must also have an appropriate balance of risk and potential benefit and the number of subjects enrolled must be capable of answering the scientific question (6).

Roth-Cline et al. enunciated the ethical principle of “scientific necessity” in pediatric research (6). This principle holds that children should not be enrolled in a clinical investigation unless it is necessary to achieve an important scientific and/or public health objective concerning the health and welfare of children. A corollary is that children should not be enrolled in studies that are duplicative or unlikely to yield important knowledge applicable to children about the product or condition under investigation.

## How Much Information to Present about the Research Study?

Increasingly, the content of consent documents is being mandated by regulation, particularly, but not only, in the USA. These regulations are weighted in favor of providing all relevant information to potential research participants. While, at one level, it is difficult to argue against this there are important consequences in that the Information/Consent Package becomes quite long, often being 20 pages or more in length. For example, The World Health Organization has published an Informed Consent Form template as a guide to investigators (7). Unfortunately, it is 11 pages in length before the investigator has added their trial-specific material.

In an Australian study, Beardsley et al. noted that in 5 years, Australian clinical trial Informed Consent information packages had increased from a median 7 pages in 2000 to 11 pages in 2005 (with a range of 7–21 pages) (8). Much of this lengthening was attributed to legislative and regulatory information imposed by Australian law, typically with patient safety in mind.

Albala et al. highlighted the inherent paradox in attempting to use consent forms to convey ever-more-complete information to potential research subjects and the subject’s ability to comprehend the information (9).

There is convincing evidence that participant comprehension decreases as the length of the document increases. Albala et al. cited data showing that consent forms that are longer than four pages are unlikely to be read (9) while Sharp has suggested that consent forms longer than 1,000 words (four double-spaced pages) are unlikely to be read (2).

There must be a balance between overwhelming potential participants with too much information and giving them insufficient information to make an informed choice. One suggested method for dealing with this paradox is to use a layered approach to the provision of information where a short readable précis is presented first before more detailed supplementary information. The National Health Service *National Research Ethics Service* in the U.K. suggests the participant information sheet be provided in two parts (10). The first part should contain brief and clear information on the essential elements of the specific study such as what the research is about and what participants will have to do. A second part should contain more detailed information.

There are ethical implications of giving people information they cannot understand and act on, particularly, when the presumed goal of that information is to enable people to make an informed choice about participation in a research study.

## How to Present and Explain Information about the Proposed Research Study?

This element is central to the process of obtaining understood consent. There are two complementary processes involved in obtaining understood consent; the presentation of the written material and, importantly, the process or methods used to explain the material such that the parent/young person or potential participant truly understands what is being presented. Research has shown that providing both written and verbal information leads to better information recall than written or verbal information alone (11).

Before exploring the detail of these two elements, it is reasonable to ask why giving someone a document to read is not, of itself, sufficient for the purposes of consent. I have already alluded to the inverse relationship between the length and complexity of the documents and comprehension. Another critical factor is health literacy.

## Impact of Health Literacy on Consent Processes

In 2007, the Australian Bureau of Statistics participated in an international comparative study – the Adult Literacy and Life-skills Survey (ALLS) (12). Health literacy is the degree to which individuals have the capacity to obtain, process and understand basic health information, and services needed to make appropriate health decisions. The survey found that around 45% of the adult population in Australia have “poor” or “very poor” health literacy skills. Another key issue is “that you can’t tell by looking” when it comes to predicting a parent’s reading skills and comprehension. Some individuals become very adept at covering up their lack of skills in this domain. Kripalani et al. suggested that subjects with low literacy be classed a vulnerable group for research participation (13).

These issues may be of little consequence for low risk non-invasive research but become highly relevant for research studies that involve more than minimal risk or research studies in children with life threatening conditions. In this situation, meticulous attention must be given to ensuring the parents truly comprehend the information before consent is taken.

Obviously these issues assume a larger dimension in the developing world where poor literacy is often widespread. Based on this information, it is reasonable to assume that many parents in a developing country would struggle to understand and appropriately analyze a consent form without additional explanation and assistance. Obtaining understood consent in these environments can be extremely challenging. There has been negligible research in this area. Bhutta emphasized the importance of senior members of the research team with capacity to answer questions being involved in the consent process and including communication experts (11).

Bhutta also addressed the issue of understood consent in developing countries and provided helpful suggestions on improving consent processes. These include providing community information sessions, a staged process for obtaining consent, using innovative materials and processes and using alternative processes for documenting consent such as making an audio recording (11).

In a comprehensive review, Campbell discusses the issue of informed consent and, in particular, the issues of *understanding* and *voluntariness* (14). Campbell observed that the understanding of research concepts affects both developed and developing settings and that the real focus should be on adapting the universal paradigms of research to local cultural norms, ideas and literacy levels (14). Emanuel et al. similarly advocate that the ethical requirements of biomedical research must be adapted to the health, economic, cultural, and technological conditions in which research is conducted (15).

## The Process of Obtaining Consent

In this overall context, the previously mentioned differences between consent, informed consent, and understood consent become crucially important. A signed consent form is documentary evidence of “consent” but gives no clue as to the process whereby the consent was obtained. Getz in a survey of recent trial participants found that one in seven participants stated that no one from the research team reviewed the consent form with them (16). Truly “understood consent” is obtained by presenting trial information in a layered manner (17) with substantial one-on-one time spent by the researcher or a nominee and the parent/young person or potential participant in which it is assumed that the person may not have the capacity to read or comprehend complex written material. “Informed consent” is a false half-way house between these two positions. The goal of all researchers in recruiting subjects should be truly “understood consent.” This is particularly so in research that involves randomization or the use of a placebo arm and, in particular if the research involves more than minimal risk.

A key element in preparing Information/Consent Packages is to ensure that the documents are written and formatted in a way that enhances their readability.

There is a growing body of literature on how best to obtain consent. Titus and Keane provided useful guidance on assessing parental understanding of informed consent (18). It was recommended that the researcher ask the potential research participant short questions after the research has been described and the consent form read, in order to assess that the potential research participant has at least a basic understanding of what the research involves. Example questions included:

o Tell me in your own words what this study is all about.
o Tell me what you think will happen to you in this study.
o What do you expect to gain by taking part in this research?
o What risks might you experience by participating in the research?
o What are your alternatives (other choices or options to participating in this research)?

The author acknowledges that this is a challenging proposition even in developed countries and that the difficulties in developing countries are much greater. The above represents an “aspirational goal” that people in developing countries need to embrace and use innovative, contextually appropriate methods to obtain consent.

The U.S. Agency for Healthcare Research and Quality published a report *Making Health Care Safer: A Critical Analysis of Patient Safety Practices* that reviewed patient safety practices and focused special attention on practices deserving of widespread implementation (19). One of these practices was: “Asking patients to recall and restate what they have been told during the informed consent process.” This was an effort to ensure that patients not only read and heard the informed consent, but more importantly, understood the informed consent.

Further, the National Quality Forum (NQF), an organization chartered to develop and implement a U.S. national strategy for healthcare quality measurement and reporting, released a report, *Safe Practices for Better Healthcare*, that endorsed healthcare practices aimed at improving patient safety throughout the healthcare system (20). One of these was that all healthcare professionals should ask patients to repeat or “teach back” what they have been told by their provider during the informed consent discussion. This report has recently been updated and includes a detailed evidence-based chapter on obtaining informed consent (21). While using the combination of written and verbal explanation is time consuming, the advantage of the “teach back” method is that areas of poor comprehension can be identified allowing further explanation to occur. Research staff may require special training in the use of the “teach back” methodology. Using the “teach back” methodology can also identify patients who, despite repeated corrective feedback, have poor comprehension of the proposed research. It is doubtful if informed consent can be obtained from such patients. The combination of providing written information along with verbal explanation and “teach back” questions should also be applied to adolescents who are being asked to assent to research participation.

Palmer et al. suggested that embedding explicit inquiries and re-explanations during the process of information disclosure serves both to model and emphasize for participants the notion that their understanding is sincerely, rather than just symbolically, desired by the researcher and their staff prior to study enrollment (22).

## Implications for Pediatric Pulmonology: The Way Forward

There are clear and specific implications of this argument for pediatric pulmonology researchers.

The concept of *understood consent* is neither new nor novel having been described for some time by Beauchamp and Childress (23). However, progress in changing the consent process toward *understood consent* has been inexorably slow.

Pediatric pulmonologists are drawn into this construct by virtue of how they obtain consent for both investigational procedures and for research studies. Recognizing that children are an inherently vulnerable group in regards to both clinical procedures and as research subjects is central to the ethical conduct of pediatric practice. The responsibility for ensuring that the child’s parents or the young person truly understands the nature of the clinical procedure or research as well as the potential risks lies with the clinician or researcher.

It is time to move from the mindset of informed consent being a signature on a page and instead embrace the ideal goal of understood consent. This will involve changes to the way both clinical and research material is prepared and presented and, in relation to research, much greater one-on-one interaction between the research team and the potential participant. The communication process must be more interactive and, ideally, include the “repeat back” methodologies described earlier. The use of culturally and developmentally appropriate alternate technologies, including posters, videos, etc. are to be encouraged as alternatives to the conventional consent Information Package. While this may be more time consuming, inclusion of these techniques will likely identify gaps in understanding to be addressed and, if the questions and responses are documented, provide an auditable record of a high standard consent process.

## Conflict of Interest Statement

The author declares that the research was conducted in the absence of any commercial or financial relationships that could be construed as a potential conflict of interest.
